# Simulation of the Membrane Process of CO_2_ Capture from Flue Gas via Commercial Membranes While Accounting for the Presence of Water Vapor

**DOI:** 10.3390/membranes13080692

**Published:** 2023-07-25

**Authors:** Daria Miroshnichenko, Maxim Shalygin, Stepan Bazhenov

**Affiliations:** A.V. Topchiev Institute of Petrochemical Synthesis, Russian Academy of Sciences (TIPS RAS), 29 Leninskiy Prospect, 119991 Moscow, Russia; dmiroshnichenko@ips.ac.ru (D.M.); mshalygin@ips.ac.ru (M.S.)

**Keywords:** CO_2_ capture simulation, flue gases, gas separation membranes, commercial membranes, mass transfer modeling

## Abstract

Carbon capture and storage is one of the potential options for reducing CO_2_ emissions from coal-fired power plants while preserving their operation. Mathematical modeling was carried out for a one-stage membrane process of carbon dioxide capture from the flue gases of coal-fired power plants using commercial gas separation membranes. Our calculations show that highly CO_2_-permeable membranes provide similar characteristics with respect to the separation process (e.g., a specific area of membrane and a specific level of electrical energy consumption) despite the significant variation in CO_2_/N_2_ and H_2_O/CO_2_ selectivity. Regarding the development of processes for the recovery of CO_2_ from flue gas using membrane technology, ensuring high CO_2_ permeance of a membrane is more important than ensuring high CO_2_/N_2_ selectivity. The presence of water vapor in flue gas provides a higher driving force of CO_2_ transfer through the membrane due to the dilution of CO_2_ in the permeate. A cross-flow membrane module operation provides better recovery of CO_2_ in the presence of water vapor than a counter-current operation.

## 1. Introduction

Despite ongoing efforts to introduce renewable energy technologies in order to replace fossil fuels, the growth in energy demand perpetuates the dominance and constancy of the share of fossil fuels (about 80%) in terms of the primary energy demand; as a result, global CO_2_ emissions continue to grow [1]. Coal-fired power plants are the largest point-source emitters of CO_2_, with thousands of plants in operation worldwide. There is a growing need to reduce their emissions as countries pursue options to meet their decarbonization goals. Carbon capture and storage is the only option allowing for a reduction in CO_2_ emissions from coal plants while preserving their operation [2,3]. There are two types of CO_2_ capture systems: pre-combustion and post-combustion capture systems. The important factors to consider when choosing a capture system are the concentration of CO_2_ in the gas stream, the pressure of the gas stream, and the type of fuel (solid or gas). The methods of gas purification from carbon dioxide can be divided into physical (physical absorption, adsorption, and membrane methods) and chemical (chemical absorption, catalytic hydrogenation, and enzymatic extraction) types.

Membrane gas separation has emerged as a compelling capture technology that offers advantages over absorption (amine-based) capture alternatives, namely, simplicity, compactness, easy scalability, environmental friendliness with no emissions, and the ability to be operated solely using electricity. Membranes are also well suited for upgrading and combination with other separation techniques. The main problem regarding the application of membrane technology for the recovery of CO_2_ from flue gas is the low pressure of the feed stream, which does not allow for the generation of a high driving force for the process. The compression of flue gas streams is economically impractical due to the very high flow rates involved (about hundreds of m^3^/s); therefore, the only solution is to apply a vacuum, sweeping, or both in the permeate side.

In [4], two simplified models of a single-stage membrane process for CO_2_ capture from power plant flue gases (13% CO_2_, 87% N_2_, no O_2_, and no H_2_O) were considered using the same membrane with equal pressure drop values. In this case, the difference lay in the driving force regime: in the first model, it was proposed that a compressor would be used to pump the initial gas flow, while in the second model, a vacuum pump on the permeate side was considered. The vacuum scheme was found to be preferable since the compressor must pump a large inlet gas stream, mainly N_2_, which significantly affects the overall level of energy consumption. The vacuum scheme only needs to pump a permeate stream, which is much lower than the feed flue gas stream. Regarding the choice of vacuum scheme, the authors calculations showed that total energy consumption is reduced by 45%, and the optimum vacuum value is 0.22 bar, while a lower permeate pressure is impractical.

**Figure 1:**
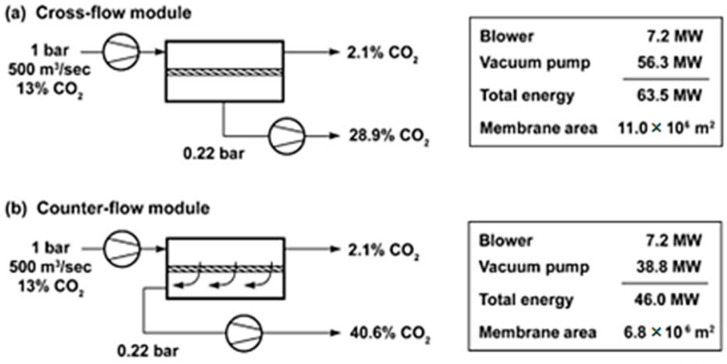
Comparison of cross-flow and counter-flow modes for flue gas separation [4].

**Figure 2:**
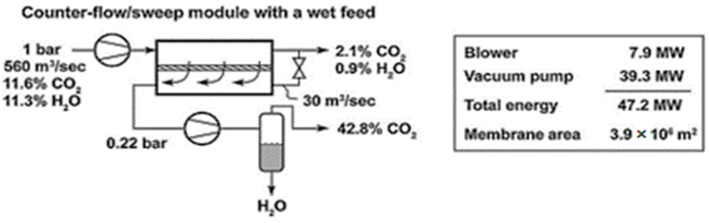
The impact of sweeping on counter-flow module performance in a wet flue gas application [4].

Based on the chosen vacuum scheme, single-stage schemes of CO_2_ extraction from the flue gases of a coal-fired power plant were considered. Calculations for cross-flow and counter-current membrane module operation modes were performed at a permeate pressure of 0.22 bar (Figure 1). To achieve a criterion of 2.1% CO_2_ in the retentate, it was found that the required membrane area needed to be 40% lower, while the required level of energy consumption need to be 28% lower for counter-current mode. Also, the counter-current mode makes it possible to achieve 40.6% CO_2_ in the permeate and 28.9% in the cross-flow mode. Nevertheless, a comparison of the different modes was only performed for the N_2_/CO_2_ mixture, and this was conducted without accounting for the presence of water vapor, whose content in flue gas is almost equal the amount of CO_2_ and can noticeably affect the characteristics of separation process. Considering the advantages of the counter-current operation, the process of CO_2_ capture in the presence of water vapor and the sweeping of permeate with part of the retentate stream was considered (Figure 2). Consequently, it was determined that the application of sweeping does not significantly affect the concentration of CO_2_ in the permeate (after water condensation), but the required membrane area drop is equal to 40%.

Calculations were carried out with regard to a membrane with a CO_2_ permeance of 1000 GPU. Despite this high permeance, the required membrane area turned out to be large (millions of m^2^), which greatly affects the capital costs.

According to an economic evaluation of CO_2_ capture [5], for membranes with a CO_2_ permeance of ~1000 GPU and a CO_2_/N_2_ selectivity ≤ 25, it is economically justifiable to use a vacuum scheme or its combination with the compression of the feed stream. For membranes with a higher CO_2_ permeance, it is preferable to use a vacuum scheme alone.

All the considered variations of the one-stage process do not allow one to achieve the 80% CO_2_ purity that has been declared to be the necessary value in the specifications stipulated by the International Energy Agency [6]. In order to comply with the specifications while using reasonable parameters for the process (in relation to the pressure of the feed and permeate streams, among other considerations), it is necessary to apply two- or multi-stage separation procedures. A study addressing this topic was presented in [7], where two-stage separation was considered using different flow patterns in combination with membrane modules to achieve 95% CO_2_ at a recovery value from 50 to 90%. It was found that the scheme concerning the supply of the first-stage permeate to the second stage was more energy efficient but required a larger membrane area. Regarding the retentate supply to the second stage, the total required membrane area is smaller, but energy consumption rose due to the need to increase pressure in order to provide the necessary CO_2_ transfer driving force. In both cases, the application of a vacuum in the permeate is more preferable than the compression of the feed stream.

There are a number of studies devoted to the analysis of CO_2_ recovery using membranes based on different materials, including biopolymers (for example, chitosan and its derivatives) or polymers, which demonstrate specific interactions with CO_2_ (facilitated transport membranes [8]). For example, a comparative analysis of the application of different membranes, including facilitated transport membranes, for CO_2_ recovery from flue gases of various sources was carried out in [9]. The properties of these materials and membranes usually have a strong dependence on water vapor activity. This peculiarity complicates the calculation of separation processes and makes the prediction of process characteristics less reliable. Therefore, such types of membranes were not considered in this work. Moreover, due to the need for millions of square meters of membrane surface to treat typical flow rates of flue gas streams, industrial scale membrane production is necessary.

This paper represents a simulation of CO_2_ recovery from power plant flue gas through a single-stage membrane process incorporating the application of a vacuum on permeate side. Calculations were carried out for a wide range of commercially produced gas separation membranes, and two modes of organizing flows in a membrane module were considered in order to assess their potential for application in this task.

## 2. Materials and Methods

### 2.1. Commercial Gas Separation Membranes: Materials and Manufacturers

In order to simulate the process of CO_2_ recovery from power plant flue gas, commercially produced polymer gas separation membranes based on polydimethylsiloxane (PDMS), poly(vinyl-trimethyl-silane) (PVTMS), polyphenylene oxide (PPO), cellulose acetate (CA), polysulfone (PSf), Tetrabromo polycarbonate (TBPC), and polyimide P84 were selected. The manufacturers and polymer materials used are presented in Table 1.

The values of the permeability coefficients and selectivity of gases and water vapor for the selected polymer materials found in the literature are presented in Table 2.

### 2.2. Modeling of Membrane Process of CO_2_ Capture from Flue Gas

**Figure 3:**
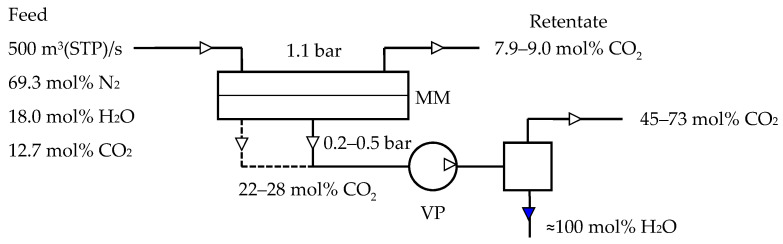
Scheme depicting the membrane process of carbon capture from the flue gases of coal-fired power plants used for modeling: solid line—cross-flow mode; dotted line—counter-current mode (C—condenser, VP—vacuum pump, and MM—membrane module).

Mathematical modeling was carried out for a one-stage membrane process (Figure 3) of carbon dioxide capture from the flue gases of coal fired power plants. Considering the compared values regarding the water vapor content in flue gas compared with that of CO_2_ along with the high membrane permeance for water vapor, a three-component mixture N_2_/CO_2_/H_2_O was considered in model. Basic models of gas transfer in the membrane module, which operates in cross-flow and counter-current modes, were considered. Description of models is represented in Appendix A.

Modeling of a one-stage membrane capture of carbon dioxide was carried out in reference to a 50% degree of CO_2_ recovery; the other parameters used in the calculation are given in Table 3. Characteristics of membranes used in modeling are given in Table 4.

## 3. Results and Discussion

The validation of the mathematical model of CO_2_ capture from flue gas was carried out via a comparison of the results of the calculations in [4] for the cross-flow and counter-current modes. The calculated membrane area was found to be 11.1 and 6.5 Mm^2^ for the cross-flow and counter-current modes, respectively, to achieve 2.1 mol% CO_2_ in the retentate. The relative deviations of the obtained membrane area from the referenced work are 0.9 and −4.6%, respectively, thus proving the model’s correctness.

Since the permeance of water vapor for the Polaris membrane has not been reported, it was estimated using a developed mathematical model and data from [4] for the case of a counter-flow/sweep module with wet feed. Estimation was carried out via the adjustment of water vapor permeance to achieve 2.1 mol% of CO_2_ and 0.9 mol% of water vapor in the retentate and 42.8 mol% of CO_2_ in the permeate after vapor condensation. The obtained permeance of water vapor for the Polaris Gen-1 membrane was found to be around 2000 GPU or 669 mol/(m^2^∙s∙kPa), which is two times higher than the permeance of CO_2_ (1000 GPU). According to the data in [24] regarding the next-generation Polaris membrane (Gen-2), the permeance of CO_2_ has risen to 2200 GPU, with a remaining degree of CO_2_/N_2_ selectivity of 50. Therefore, the permeance of water vapor for the Polaris Gen-2 membrane was estimated to be two times higher than the permeance of CO_2_ (similar to Polaris Gen-1), which is 4400 GPU or 1470 mol/(m^2^∙s∙kPa).

**Figure 4:**
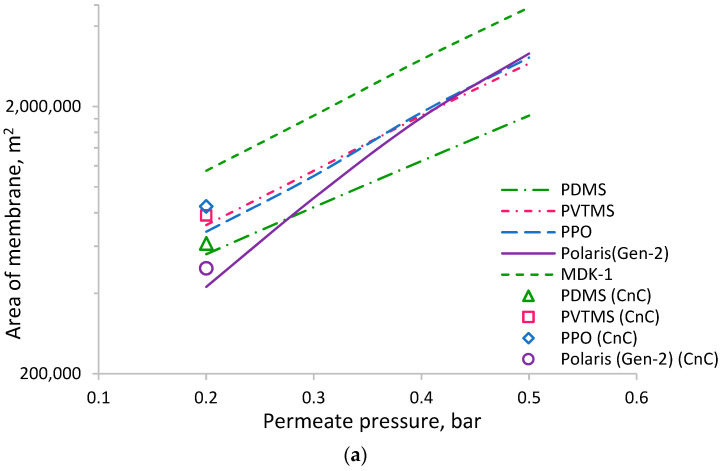

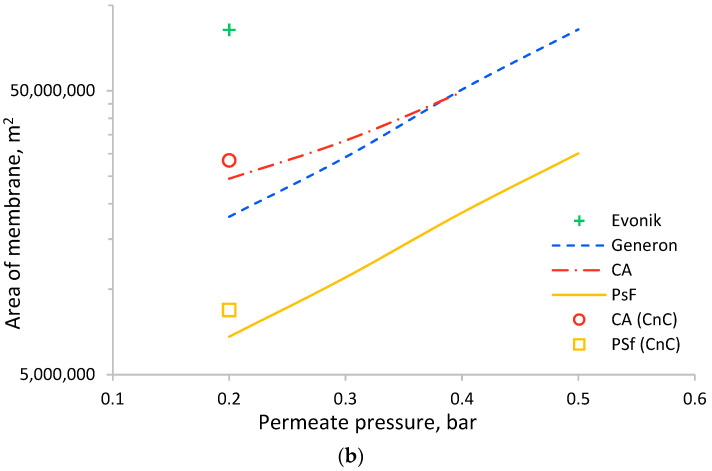
The dependence of membrane area ((**a**) high permeance membranes for CO_2_, (**b**) low permeance membranes for CO_2_) on permeate pressure for achieving a recovery of 50% of CO_2_: lines—CF mode; markers—CnC mode.

The dependences of the required membrane area on the permeate pressure in the range of 0.2–0.5 bar (Figure 4a,b) were calculated with respect to achieving a 50% degree of CO_2_ recovery. Increasing the permeating pressure required a larger area of all the membranes due to the decrease in the driving force of CO_2_ permeation (the difference between component partial pressures in the upstream and downstream regions). Among the membranes considered in Figure 4a, the PDMS-based membrane provides the smallest area and the MDK-1 membrane provides the largest area at low permeate pressure. The obtained values are chiefly influenced by the permeance of CO_2_, which is 1.8 times lower for the MDK-1 membrane compared to the membranes based on PDMS, PVTMS, and PPO. Figure 4b shows the calculated dependencies for a group of membranes with much lower CO_2_ permeance, namely, the CA-, PSf-, TBPC-, and P84-based membranes (for the P84-based membrane (Evonik), only a one-point reduction in permeate pressure was calculated). The required membrane area for this group is one to two orders of magnitude higher compared to the first group of membranes.

**Figure 5:**
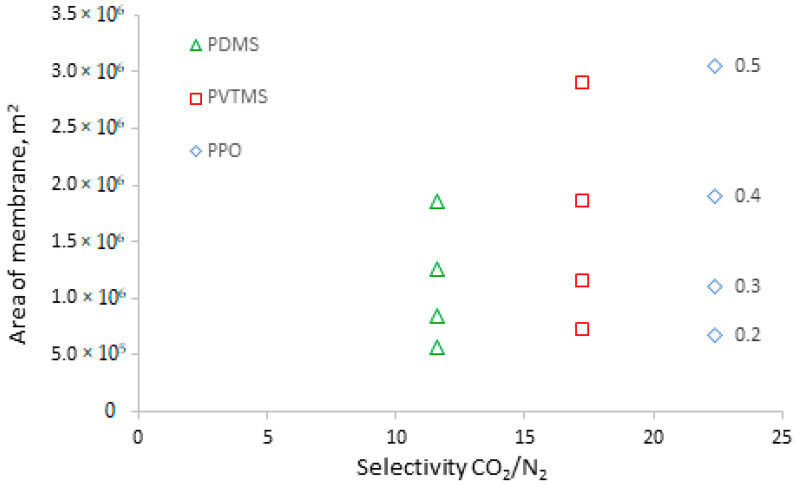
The dependence of membrane area on the selectivity of CO_2_/N_2_ for achieving 50% CO_2_ recovery at different permeate pressures (numbers near markers).

**Figure 6:**
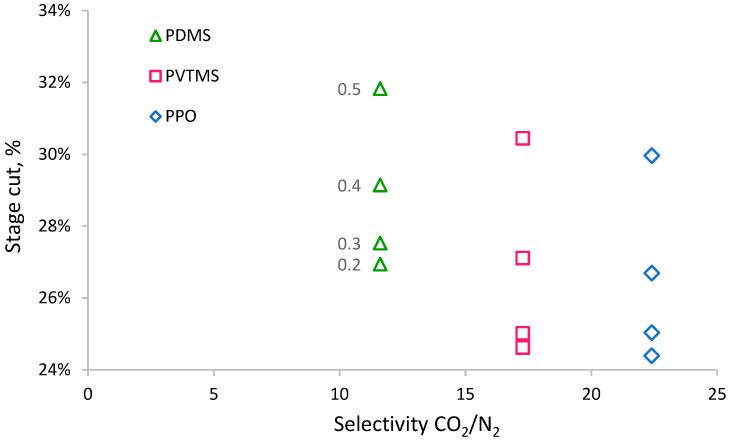
The dependence of stage cuts on the selectivity of CO_2_/N_2_ for achieving 50% CO_2_ recovery at different permeate pressures (numbers near markers).

Membranes based on PVTMS and PPO have a permeance of CO_2_ similar to the PDMS-based membrane; nevertheless, the values of the required membrane area for these membranes vary significantly. Such behavior can be explained by the influence of the permeance of other components (N_2_ and H_2_O). Due to the low value of the feed-to-permeate pressure ratio, the higher permeability of nitrogen and water vapor in the PDMS-based membrane helps to dilute CO_2_ in the permeate, thereby reducing its partial pressure and increasing the driving force for its transfer; therefore, the required membrane area is lower. Such a situation leads to a counter-intuitive conclusion that lower-selectivity CO_2_/N_2_ and CO_2_/H_2_O (and a higher permeance of the membrane for N_2_ and H_2_O) provide better recovery of CO_2_. Figure 5 shows the dependence of membrane area on CO_2_/N_2_ selectivity for membranes with almost similar CO_2_ permeances (PDMS, PVTMS, and PPO). Higher membrane selectivity results in a more rapid increase in membrane area at a higher permeate pressure. If selectivity changes from 11.6 (for PDMS) to 22.4 (for PPO) at a permeate pressure of 0.2 bar, the increase in membrane area is insignificant, while at a permeate pressure of 0.5 bar, the increase in membrane area is around 1.6-fold. Thus, lower selectivity can provide a benefit in terms of the required membrane area (CAPEX) depending on the chosen process conditions. At the same time, lower selectivity leads to the necessity of higher stage cuts to achieve the required degree of CO_2_ recovery (Figure 6); for example, a selectivity change from 11.6 to 22.4 (for PPO) requires a decrease in stage cuts of around 2.5 and 1.8% at permeate pressures of 0.2 and 0.5 bar, respectively. A lower stage cut results in a demand for lower productivity of the vacuum pump and lower power consumption (CAPEX and OPEX, respectively).

The dependencies for the PVTMS- and PPO-based membranes are very close to each other (Figure 4a) because of the similar permeances of CO_2_ (322 and 380 mol/(m^2^∙s∙kPa), respectively) and N_2_ (18.6 and 16.9 mol/(m^2^∙s∙kPa), respectively) despite the considerable difference in H_2_O permeance (see Table 4).

**Figure 7:**
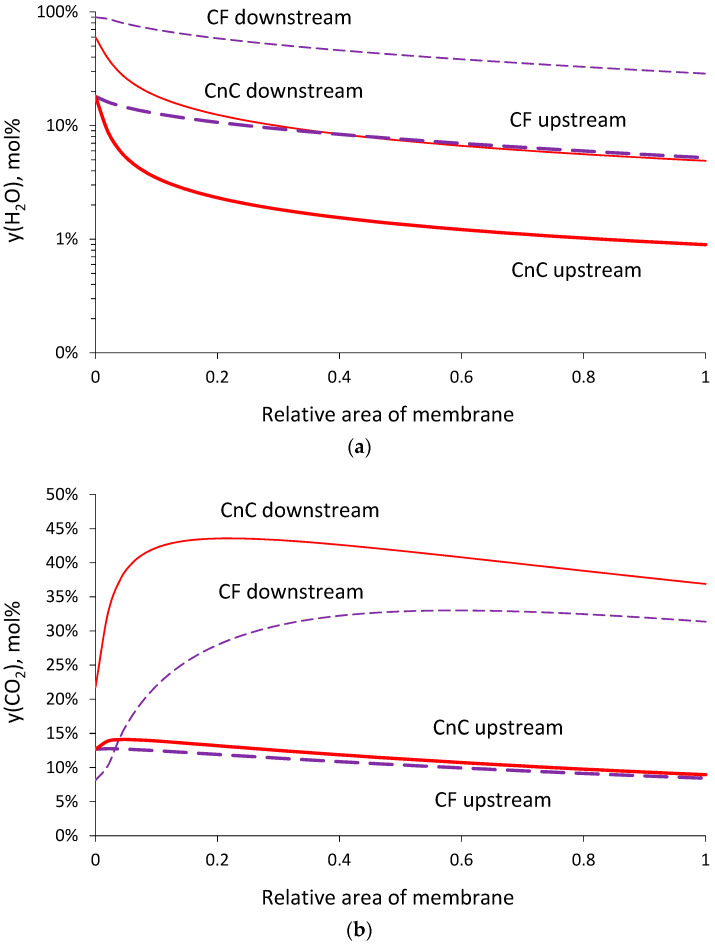
Concentration profiles of water vapor (**a**) and CO_2_ (**b**) upstream and downstream of membrane module under CF and CnC operation modes (example for PPO membrane: permeate pressure of 0.2 bar; 50% CO_2_ recovery).

In the case of low permeate pressure (0.2 bar), the required membrane area was also calculated for the CnC mode. The obtained values of area appear to be higher than those for the CF mode for all the considered membranes (markers in Figure 4). Such behavior can be explained by the influence of water vapors on the driving force of CO_2_ transfer. The corresponding concentration profiles of water vapor and CO_2_ in the upstream and downstream for the CF and CnC modes of membrane module operation are presented in Figure 7. For the CF mode, the water vapor concentration profile upstream is more “flat” (Figure 7a), leading to high water vapor flux through the membrane over the entire area and supporting the dilution of CO_2_ in the permeate by water vapor, which is especially important near the retentate outlet, where the CO_2_ content upstream is low (Figure 7b). Regarding the CnC mode, water vapor recovered more efficiently, and its content in the upstream decreased much faster compared to the CF mode, which did not aid in the dilution of CO_2_ in the permeate near the retentate outlet to provide higher driving force and better CO_2_ recovery. Considering such behavior, further calculations were made solely for the CF mode.

**Figure 8:**
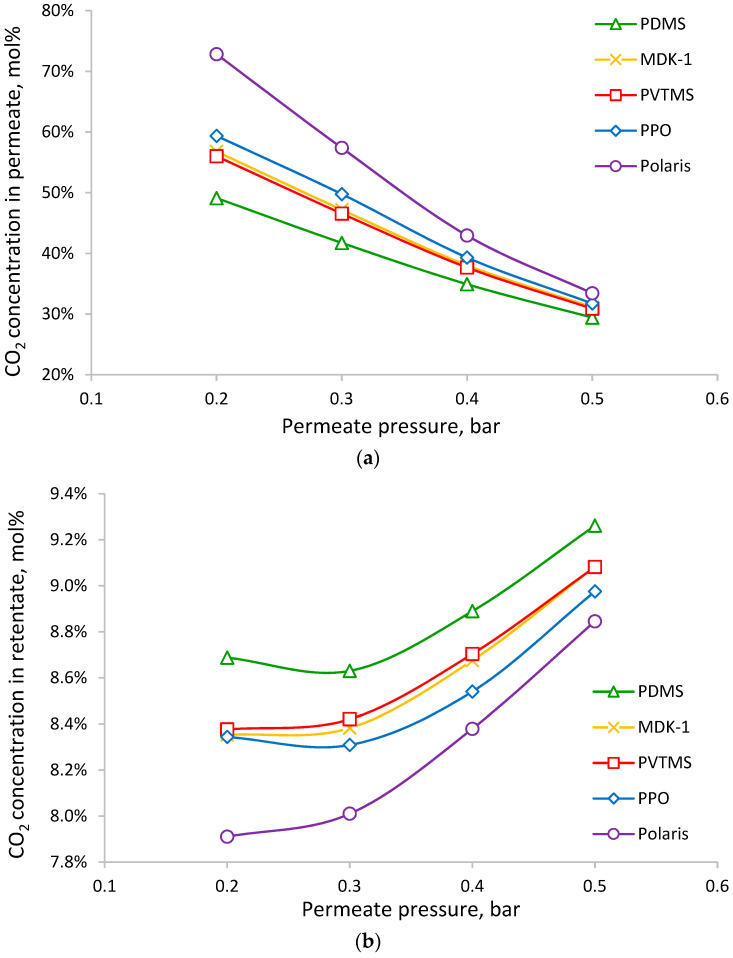
The dependence of CO_2_ molar fraction in permeate (**a**) and retentate (**b**) on permeate pressure for achieving 50% CO_2_ recovery.

The obtained dependencies of the CO_2_ molar fraction in the permeate (after the condensation of water vapor) and the retentate on permeate pressure are shown in Figure 8. At low permeate pressure, the selectivity of the membrane has a significant influence on the CO_2_ concentration in the permeate. The Polaris membrane provides 73 mol% of CO_2_, whereas the PDMS membrane only provides 49 mol% of CO_2_. The absolute values and differences become less noticeable at higher permeate pressure; for example, at 0.5 bar, the concentration of CO_2_ in the permeate is 33 and 29 mol% for the Polaris and PDMS membranes, respectively. The behaviors of the dependencies of the CO_2_ concentration in the retentate are opposite, but the effect of the selectivity of the membrane is relatively weak: the entire range of CO_2_ molar fraction variation in the retentate is 7.9–9.3 mol%.

**Figure 9:**
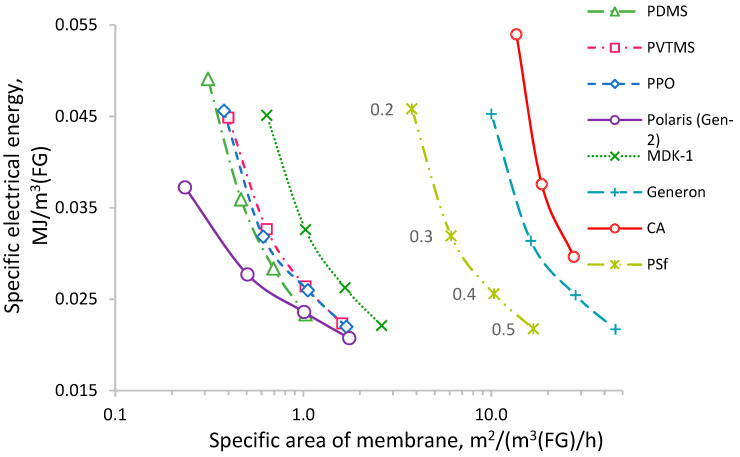
Dependence of specific electrical energy consumption on the specific area of membrane with varying permeate pressure 0.2–0.5 bar (numbers near markers) to achieve 50% CO_2_ recovery.

An estimated calculation of specific energy was carried out considering the required power for a vacuum pump as a main consumer. The calculated values were paired with the corresponding relative area of membrane for a given pressure of a permeate. The obtained dependencies (Figure 9) can be used as the basis for a preliminary economic assessment for the estimation of the OPEX and CAPEX for the considered process.

The dependencies are monotonous in all cases and show an increase in the specific energy consumption as the area of membrane decreases, which is caused by a decrease in permeate pressure. For a more accurate economic assessment (RUB/tonne (CO_2_)), it is necessary to consider the cost of electricity and the cost of a unit of an installed membrane area for a given membrane (including the cost of membrane modules, pipes, taps, valves, etc., as well as the installation cost).

This study does not discuss all issues regarding the application of gas separation membranes for CO_2_ recovery from flue gases. For example, the considered membranes are mostly composed of hollow fibers that, in real applications, may lead to the appearance of problems such as a noticeable pressure drop in the upstream (additional energy consumption) and the downstream (a loss of vacuum) or the capillary condensation of water vapor inside hollow fibers. The stability of membrane materials under process conditions in the presence of typical impurities in flue gas also demands further research as it was first conducted by the MTR company.

## 4. Conclusions

Our calculations show that highly permeable membranes provide similar indicators of the separation process despite the significant variation in CO_2_/N_2_ and H_2_O/CO_2_ selectivity. Among the highly permeable membranes, it was determined that the lowest required membrane area corresponded to the PDMS-, PVTMS-, and PPO-based membranes and Polaris Gen-1. Considering the development of more permeable second- and third-generation Polaris membranes, these membranes will demonstrate almost absolute superiority over other commercial membranes. Regarding the development of the CO_2_ recovery process from flue gas using membrane technology, the high CO_2_ permeance of a membrane is more important than high CO_2_/N_2_ selectivity. The presence of water vapor in flue gas provides higher CO_2_ transfer through the membrane due to the dilution of CO_2_ in the permeate. The cross-flow mode shows more effective CO_2_ recovery than the counter-current mode because of the flatter concentration profile of the water vapor over the membrane, which promotes the dilution of CO_2_ in the permeate.

## Figures and Tables

**Table 1:** Available data regarding manufacturing companies and materials used in the production of commercial gas separation membranes.

Manufacturer	Commercial Name	Polymer	Ref.
STC “Vladipor”, Vladimir, Russia	MDK-1	PDMS-based copolymer	[10]
NPO PJSC “Cryogenmash”, Balashikha, Russia	PVTMS	PVTMS	[11]
Parker Hannifin, Cleveland, OH, USA	Parker	PPO	[12]
UOP (A Honeywell Company), Charlotte, NC, USA Schlumberger, Houston, TX, USA	Separex, Cynara	CA	[12]
Air products, Allentown, PA, USA	Prizm	PSf	[12]
MTR, 39630 Eureka Dr, Newark, CA, USA	Polaris Gen-2	-	[12]
Generon, 16250 Tomball Parkway Houston, TX, USA	Generon	TBPC	[12]
Evonik, Essen, Germany	Sepuran	P84	[13]

**Table 2:** Available data on CO_2_, N_2_, and water vapor permeability coefficients for membrane polymers.

Polymer	P (CO_2_), Barrer	P (N_2_), Barrer	P (H_2_O), Barrer	*α* (CO_2_/N_2_)	*α* (H_2_O/N_2_)	Ref.
PDMS	3250	280	36,000	11.6	129	[14]
PVTMS	190	11.0	1450	17.3	132	[15]
PPO	56.0	2.50	4060	22.4	1620	[16,17]
CA	2.40	0.250	6800	9.60	27,200	[16,18]
PSf	5.60	0.250	2000	22.4	8000	[16,19]
TBPC	4.23	0.182	795	23.2	4370	[20]
P84	1.20	0.0240	1840	50.0	2080	[21]

**Table 3:** The basis for the simulation of a membrane process for CO_2_ recovery from flue gases.

Parameters	Values
Flue gas feed flow rate, m^3^ (STP)/h	1,800,000
Initial flue gas composition, mol%:	
N_2_	69.3
CO_2_	12.7
H_2_O	18.0
Feed pressure (absolute), bar	1.1
Permeate pressure (absolute), bar	0.2–0.5
Temperature, °C	40

**Table 4:** Permeances of membranes used in calculations.

Polymer Membrane	Selective Layer Thickness, μm	Q (CO_2_)∙10^6^, mol/(m^2^∙s∙kPa)	Q (N_2_)∙10^6^, mol/(m^2^∙s∙kPa)	Q (H_2_O)∙10^6^, mol/(m^2^∙s∙kPa)
PDMS	3.0	362	31.2	4010
MDK-1 *	n/a	203	11.2	2040
PVTMS	0.2	318	18.4	2430
PPO	0.05	375	16.7	27,200
CA	0.10	7.99	0.790	22,700
PSf	0.05	37.5	1.70	13,400
Polaris Gen-2	n/a	737	14.8	1470 **
TBPC	0.10	13.8	0.590	2660
P84	0.10	4.05	0.0790	6270

* Calculated in [22,23]. ** Calculated in [4,24]. n/a—not available.

**Figure A1:**
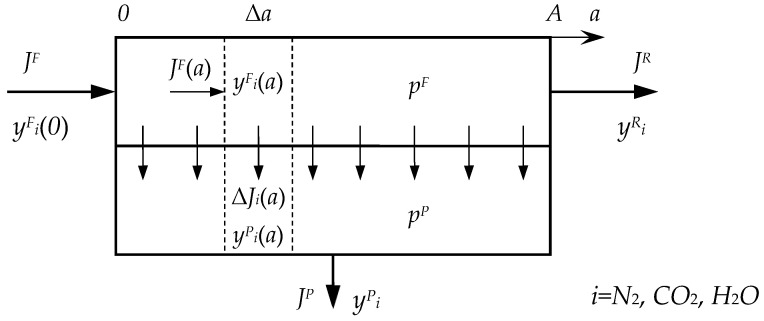
Scheme of cross-flow mode of mass transfer calculation in the membrane module used in the modeling.

**Figure A2:**
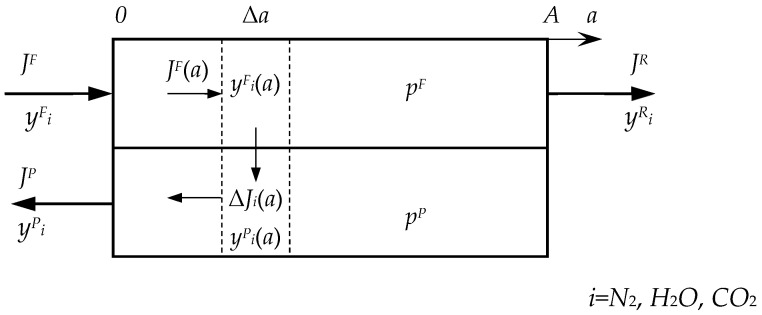
Scheme of counter-current mode of mass transfer calculation in the membrane module used in the modeling.

## Data Availability

Not applicable.

## Nomenclature

*A*	area of membrane, m^2^
*a*	coordinate of membrane area, m^2^
*J*	flow rate, mol/s
*P*	permeability coefficient, barrer
*p*	pressure, bar
*Q*	permeance, mol/(m^2^∙s∙kPa)
*y*	molar fraction, mol%
Greek letter	
α	selectivity of polymer/membrane
θ	stage cut, %
θ_i_	recovery of component i, %
Superscript	
0	initial value
F	feed
P	permeate
R	retentate
Subscript	
i	component

## Appendix A

The mathematical model includes the following assumptions: isothermal conditions; plug flow in feed membrane module channel; permeate is drained from the membrane without mixing between surrounding regions for the cross-flow mode or plug flow in the permeate membrane module channel for the counter-current mode; and gas permeance is independent of feed gas composition and process conditions (Figure A1 and Figure A2).

Cross-flow mode:

(A1) $$\left\{ \begin{array}{l} \Delta J_{i}^{F}(a)=-Q_{i}\left(p_{}^{F}y_{i}^{F}(a)-p_{}^{P}y_{i}^{P}(a)\right)\Delta a\\ J_{i}^{P}(a)=Q_{i}\left(p_{}^{F}y_{i}^{F}(a)-p_{}^{P}y_{i}^{P}(a)\right)\Delta a\\ y_{i}^{F}(a)=\frac{J_{i}^{F}(a)}{\sum J_{i}^{F}(a)}\\ y_{i}^{P}(a)=\frac{J_{i}^{P}(a)}{\sum J_{i}^{P}(a)}\\ J_{i}^{F}(0)=J_{}^{F}y_{i}^{0} \end{array}\right.$$

Counter-current mode:

(A2) $$\left\{ \begin{array}{l} \Delta J_{i}^{F}(a)=-Q_{i}\left(p_{}^{F}y_{i}^{F}(a)-p_{}^{P}y_{i}^{P}(a)\right)\Delta a\\ \Delta J_{i}^{P}(a)=Q_{i}\left(p_{}^{F}y_{i}^{F}(a)-p_{}^{P}y_{i}^{P}(a)\right)\Delta a\\ y_{i}^{F}(a)=\frac{J_{i}^{F}(a)}{\sum J_{i}^{F}(a)}\\ y_{i}^{P}(a)=\frac{J_{i}^{P}(a)}{\sum J_{i}^{P}(a)}\\ J_{i}^{F}(0)=J_{}^{F}y_{i}^{0}\\ J_{i}^{P}(A)=0 \end{array}\right.$$

The system of equations was solved numerically using the finite difference method.

Stage cut and CO_2_ recovery were calculated using the following equations:

(A3) $$\theta =\frac{J_{}^{P}}{J_{}^{F}}$$

(A4) $$\theta_{CO_{2}}^{}=\frac{J_{}^{P}y_{CO_{2}}^{P}}{J_{}^{F}y_{CO_{2}}^{F}}$$
